# Increased synthesis and deposition of extracellular matrix proteins leads to endoplasmic reticulum stress in the trabecular meshwork

**DOI:** 10.1038/s41598-017-14938-0

**Published:** 2017-11-02

**Authors:** Ramesh B. Kasetti, Prabhavathi Maddineni, J. Cameron Millar, Abbot F. Clark, Gulab S. Zode

**Affiliations:** The North Texas Eye Research Institute, University of North Texas Health Science Center at Fort Worth, TX 76107 Fort Worth, USA

## Abstract

Increased synthesis and deposition of extracellular matrix (ECM) proteins in the trabecular meshwork (TM) is associated with TM dysfunction and intraocular pressure (IOP) elevation in glaucoma. However, it is not understood how ECM accumulation leads to TM dysfunction and IOP elevation. Using a mouse model of glucocorticoid (GC)-induced glaucoma, primary human TM cells and human post-mortem TM tissues, we show that increased ECM accumulation leads to endoplasmic reticulum (ER) stress in the TM. The potent GC, dexamethasone (Dex) increased the secretory protein load of ECM proteins in the ER of TM cells, inducing ER stress. Reduction of fibronectin, a major regulator of ECM structure, prevented ER stress in Dex-treated TM cells. Overexpression of fibronectin via treatment with cellular fibronectin also induced chronic ER stress in primary human TM cells. Primary human TM cells grown on ECM derived from Dex-treated TM cells induced ER stress markers. TM cells were more prone to ER stress from ECM accumulation compared to other ocular cell types. Moreover, increased co-localization of ECM proteins with ER stress markers was observed in human post-mortem glaucomatous TM tissues. These data indicate that ER stress is associated with increased ECM accumulation in mouse and human glaucomatous TM tissues.

## Introduction

Glaucoma is the second leading cause of irreversible vision loss affecting about 70 million people worldwide, and is more prevalent in African Americans^1^. Primary open angle glaucoma (POAG), the most common form of glaucoma is characterized by progressive loss of retinal ganglion cell (RGC) axons and irreversible loss of vision^2^. Although the exact cellular mechanisms that cause glaucoma are poorly understood, elevated intraocular pressure (IOP) is a major associated risk factor^3^. The maintenance of IOP in a narrow range is essential for survival of the neuroretina. The trabecular meshwork (TM), a specialized tissue located at the iridocorneal angle of the eye, maintains normal IOP by regulating aqueous humor outflow resistance. In POAG, there is increased resistance to aqueous humor outflow through the TM, thus elevating IOP^3–5^. Although glaucomatous TM damage is associated with various morphological and biochemical changes including extracellular matrix (ECM) protein accumulation^6–12^ and loss of TM cells^13^, the exact pathological mechanisms that lead to this glaucomatous TM damage are not fully understood.

Glucocorticoids (GCs) are the mainstream treatment for the plethora of inflammatory disorders including ocular conditions such as dry eye, allergic eye disease, inflammation following eye surgery, uveitis, diabetic macular edema and many others^14^. Ocular hypertension is a serious side effect of glucocorticoid therapy. Topical or systemic GC administration can lead to ocular hypertension in about 30–50% patients depending on the route of administration, and sustained GC treatment can lead to secondary iatrogenic open-angle glaucoma if GCs are not withdrawn^15–17^. Although GC-induced glaucoma is a form of secondary iatrogenic open-angle glaucoma, its clinical presentations are similar in many ways to POAG^16^. Furthermore, GC responsiveness is significantly higher in POAG patients^18^. Similar to POAG, GC-induced glaucoma is also caused by increased resistance to aqueous humor outflow at the TM^16,19^. Therefore, a large number of *in vitro* and *in vivo* studies have examined GC-induced ocular hypertension to better understand glaucomatous TM damage^16,20,21^. GCs exhibit diverse effects on the TM including altered TM gene and protein expression, reduced TM phagocytic function, increased ECM synthesis and deposition as well as changes in the TM cytoskeleton^15,16,21–27^. GC-induced ECM deposition in the TM is of great research interest because similar changes in ECM were observed in POAG patients^12^. Increased levels of fibronectin^7^, collagen VI^8^, and transglutaminase (TGM) 2^28^ were shown in the TM of POAG patients. Increased fibril-like material deposits were observed in human eyes treated with GCs^23,29^. Numerous other studies have shown that the potent GC, dexamethasone (Dex), increased ECM deposition including fibronectin, laminin, elastin and type IV collagen in TM cells^30–33^. However, it is not understood how ECM accumulation leads to TM dysfunction and IOP elevation.

GC-induced ocular hypertension has been reported in a number of species^14^ including monkeys^34^, rabbits^35^, rats^36^, cows^37^, sheep^38^, cats^39^ and mice^40–42^. In addition, effects of GCs on the TM have been studied using primary human TM cells and *ex-vivo* bovine or human anterior segment perfusion systems^20,43^. Recently, we have demonstrated that topical ocular or periocular injections of Dex in C57BL/6J mice elevates IOP^44,41^. Using this mouse model of Dex-induced glaucoma, we showed that Dex induces endoplasmic reticulum (ER) stress in the TM, which is associated with Dex-induced IOP elevation. Since ECM proteins are synthesized and processed in the ER, the increased secretory load due to Dex in specialized TM cells may exceed the normal ER capacity, thereby inducing ER stress. Not all synthesized ECM proteins are properly folded and to cope up with protein unfolding, cells activate a protective unfolded response (UPR) pathway. UPR activates the downstream signaling proteins including ER chaperones (GRP78 and GRP94) and induces ER-associated degradation (ERAD) via proteasomes to normalize ER homeostasis^45–47^. Under chronic and persistent ER stress, UPR can also activate cell dysfunction/death via ATF4/CHOP^48^. Although TM cells may activate protective UPR pathway that normally aids in protein folding and proteosomal degradation of misfolded proteins, we hypothesize that TM cells are not well equipped to handle excessive secretory protein load, inducing chronic ER stress. These events can in turn activate apoptotic signals^46^. The UPR signal transduction pathway regulated by ATF4 and CHOP were significantly increased in Dex treated eyes and in post-mortem human glaucomatous TM tissues^49,50^. The purpose of this study was to determine whether excessive ECM accumulation leads to ER stress in TM cells. In addition, we examined whether ECM accumulation is associated with ER stress in human glaucomatous TM tissues.

## Results

### Dex-induced IOP elevation is associated with reduced outflow facility and abnormal ECM accumulation in the TM

**Figure 1:**
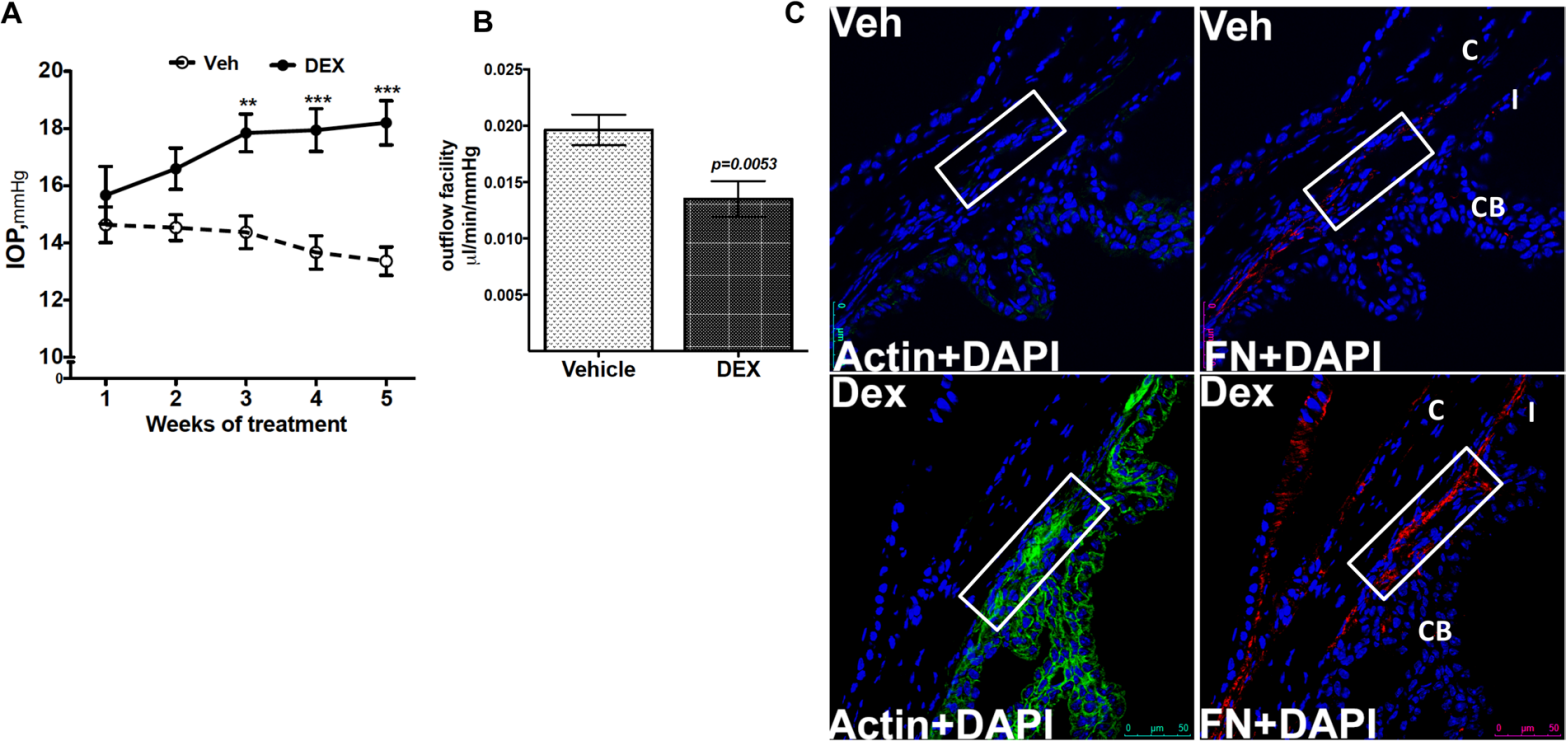
Dex-induced IOP elevation is associated with reduced outflow facility and increased ECM accumulation in the TM. (**A**) Elevated IOP in Dex-treated mice. Topical ocular Dex (0.1%) or Veh (PBS) eye drops were applied to A/J mice 3 times a day for 5 weeks, and IOPs were recorded weekly. A significant IOP elevation was observed starting from 3-weeks of Dex treatment (n =  10 each group, 2-WAY ANOVA, **P < 0.01, ***P < 0.001). (**B**) Reduced outflow facility in Dex-treated mice. A significant reduction in outflow facility was observed in 7-weeks Dex-treated mice compared to Veh-treated mice (n = 8 each group, unpaired student’s t-test). (**C**) Dex increased fibronectin and actin immunostaining in the TM of 7-weeks Dex-treated mice (n = 3) compared with Veh-treated mice (n = 3). White box indicates TM. CB = ciliary body, I = Iris, C = cornea. Scale bar = 50 μm.

**Figure 2:**
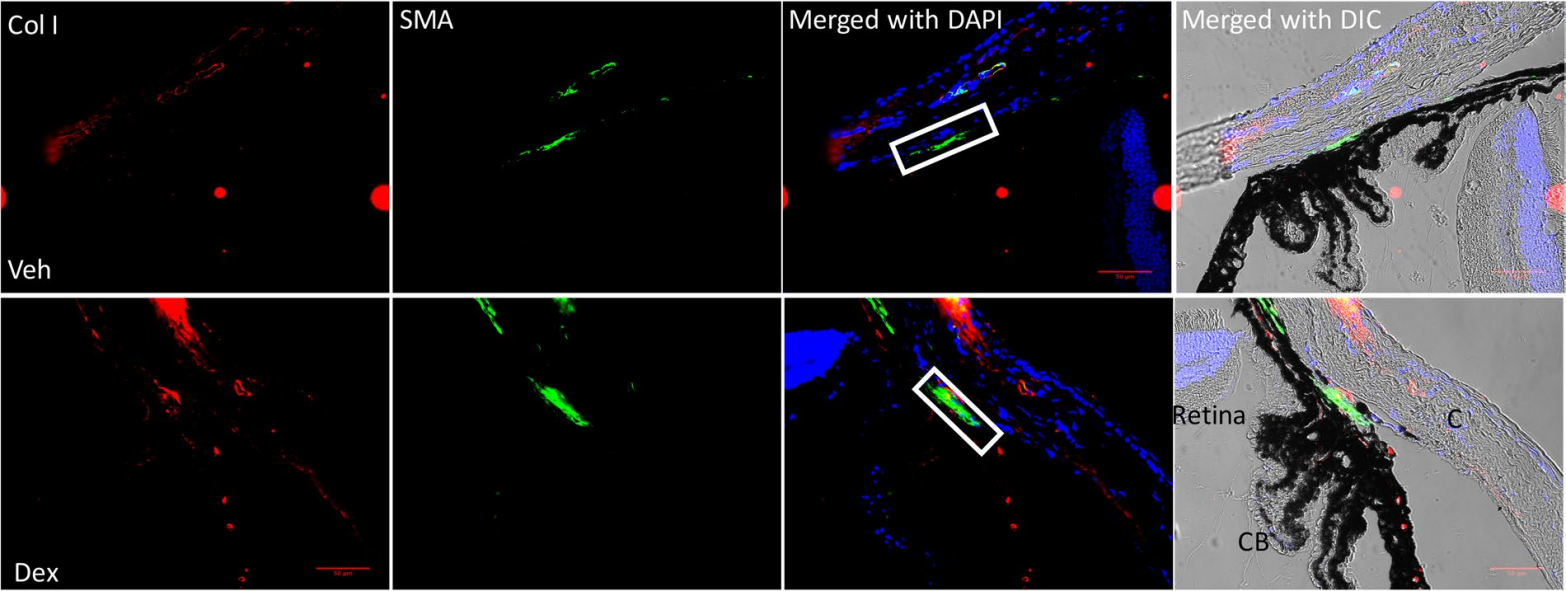
Dex increases Col I in the TM: Dex increases Col I and α-smooth muscle actin in the TM of Dex-treated mice (n = 3) compared with Veh-treated mice (n = 3). White box indicates TM. CB = ciliary body, I = Iris, C = cornea. Scale bar = 50 μm.

We first examined whether Dex treatment elevates IOP by increasing outflow resistance at the TM. As shown in Fig. 1A, topical ocular Dex treatment leads to sustained and significant IOP elevation starting from 3 weeks of treatment. The approximate increase of IOP in Dex-treated mice over Veh-treated mice was 1.0 mmHg at 1-week (*P* > *0.05*), 2.0 mmHg at 2 weeks (*P* > *0.05*), 3.5 mmHg at 3 weeks (*P* < *0.01*), 4.2 mmHg at 4 weeks (*P* < *0.001*) and 4.8 mmHg at 5 weeks of treatment (*P* < *0.001*, n = 10, 2-way ANOVA). Using a constant flow infusion method, we measured outflow facilities in Veh and Dex-treated mice (Fig. 1B). A significant decrease (*p* = *0.0053*) in outflow facility was observed in Dex-treated mice (0.0135 ± 0.001581 µl/min/mmHg) compared to Veh-treated mice (0.01963 ± 0.001349 µl/min/mmHg). There was ~30% decrease in outflow facility in Dex-treated mice compared to Veh-treated mice. Several studies have shown that Dex induces ECM deposition and actin changes in the TM^16,21,23,51^. To confirm this, we next examined whether Dex treatment leads to increased ECM deposition and actin changes in the TM of our mouse model of Dex-induced ocular hypertension (Fig. 1C). Immunostaining for fibronectin and actin (Fig. 1C) as well as collagen type I (Col I; Fig. 2) in Veh and Dex-treated mice demonstrated that Dex prominently increased fibronectin, actin and Col I in mouse TM tissues.

### Dex increases secretory protein overload including ECM proteins in the ER of TM cells

**Figure 3:**
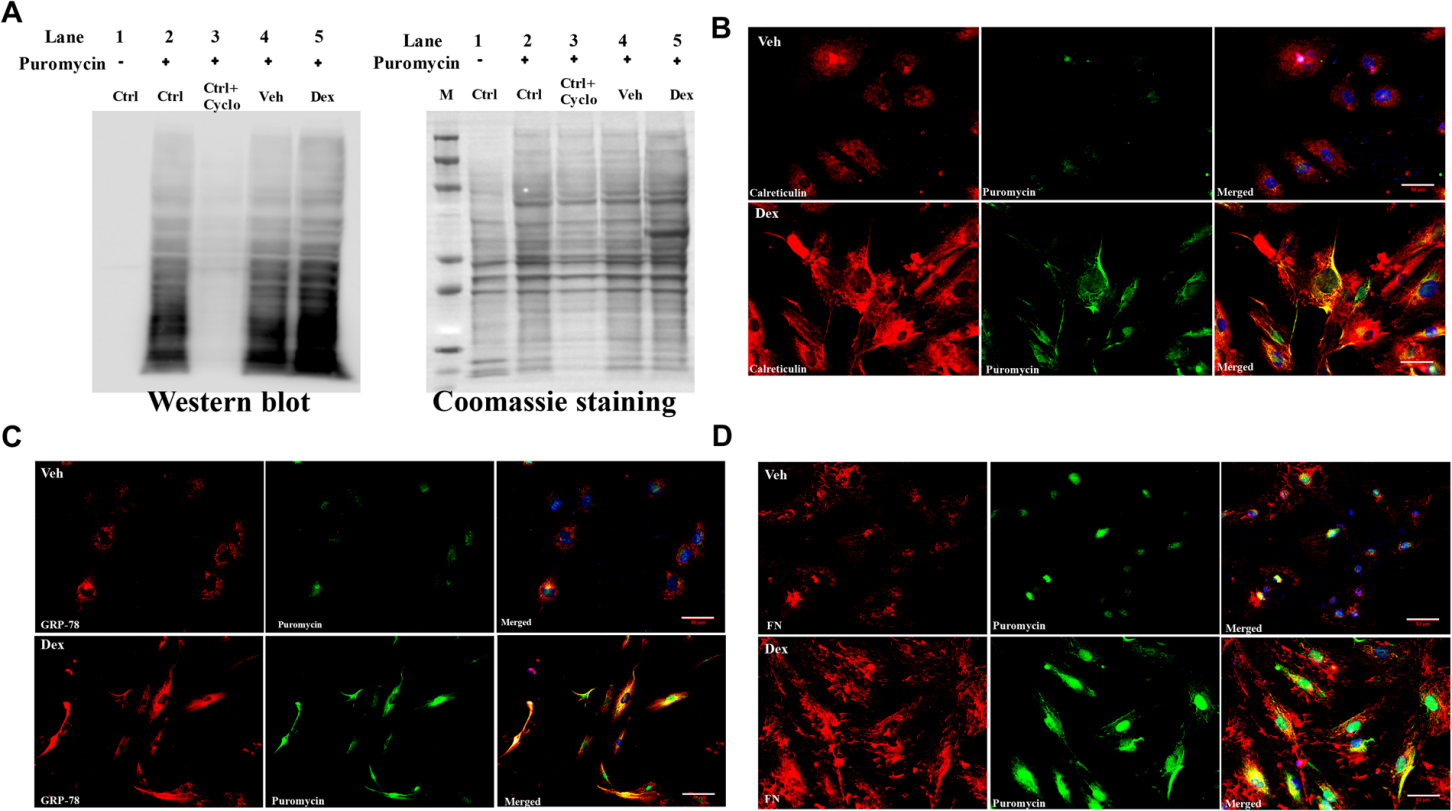
Dex increased general protein synthesis and induced secretory protein overload including ECM proteins in the ER of TM cells. (**A**) GTM3 cells were treated with Veh or Dex for 3 days and incubated with puromycin for 30 min. Western blot analysis using puromycin antibody demonstrated that Dex increased de novo protein synthesis. Lane 1; cells without puromycin (negative control), lane 2; cells treated with puromycin alone, lane 3; cells treated with cyclohexamide and puromycin. Lane 3 is compared with lane 2 to show that protein synthesis inhibitor reduces puromycin labeling of newly synthesized proteins. Lanes 4 and 5 are treated with Veh or Dex along with puromycin. Coomassie staining was performed to view total proteins. Experiments were run thrice in GTM3 cell line. M = protein ladder. (**B**–**D**) Primary human TM cells (n = 2 cell strains) were treated with Veh or Dex for 3 days and incubated with puromycin for 30 min. Co-localization studies were performed using immunostaining for puromycin antibody along with ER marker, calreticulin (**B**) or ER stress marker, GRP78 (**C**) or fibronectin (**D**).

To determine whether increased ECM synthesis induced by Dex in TM cells exceeds the normal ER capacity, thereby inducing ER stress, we first examined whether Dex increases de novo protein synthesis using the SUnSET assay, a nonradioactive method of protein synthesis^52^. In this assay, puromycin, a structural analog of aminoacyl tRNAs gets incorporated into the nascent polypeptides and stops elongation. At lower concentrations, puromycin incorporation in newly synthesized proteins reflects the rate of de novo protein synthesis. GTM3 cells were treated with Veh or Dex for 3 days and incubated with puromycin for 30 min. Western blot analysis using a puromycin antibody revealed that Dex increased general protein synthesis in TM cells (Fig. 3A). Coomassie staining was used as a total protein loading control. As a negative control, TM cells incubated without puromycin demonstrated no puromycin reactive bands in the Western blot (Fig. 3A, lane 1). As a positive control, TM cells incubated with the protein synthesis inhibitor, cycloheximide reduced protein synthesis (lane 3) compared to control cells incubated with puromycin (lane 2). Compared to Veh-treated TM cells, Dex increased protein synthesis as evident from the intense band at the bottom of gel representing puromycin truncated proteins (lane 4 Vs lane 5). We next examined whether Dex increases the secretory protein load in the ER. Primary human TM cells (n = 2 cell strains) were treated with Veh or Dex for 3 days and incubated with puromycin for 30 min. Fixed cells were immunostained with a puromycin antibody and the ER marker calreticulin to examine de novo protein synthesis of secretory proteins in the ER. Immunostaining demonstrated that Dex dramatically increased the protein synthesis of secretory proteins from the ER of TM cells (Fig. 3B). Moreover, immunostaining for puromycin and GRP78 demonstrated that de novo protein synthesis is associated with ER stress (Fig. 3C). Immunostaining for puromycin and fibronectin demonstrated that Dex induces de novo synthesis of fibronectin (Fig. 3D).

**Figure 4:**
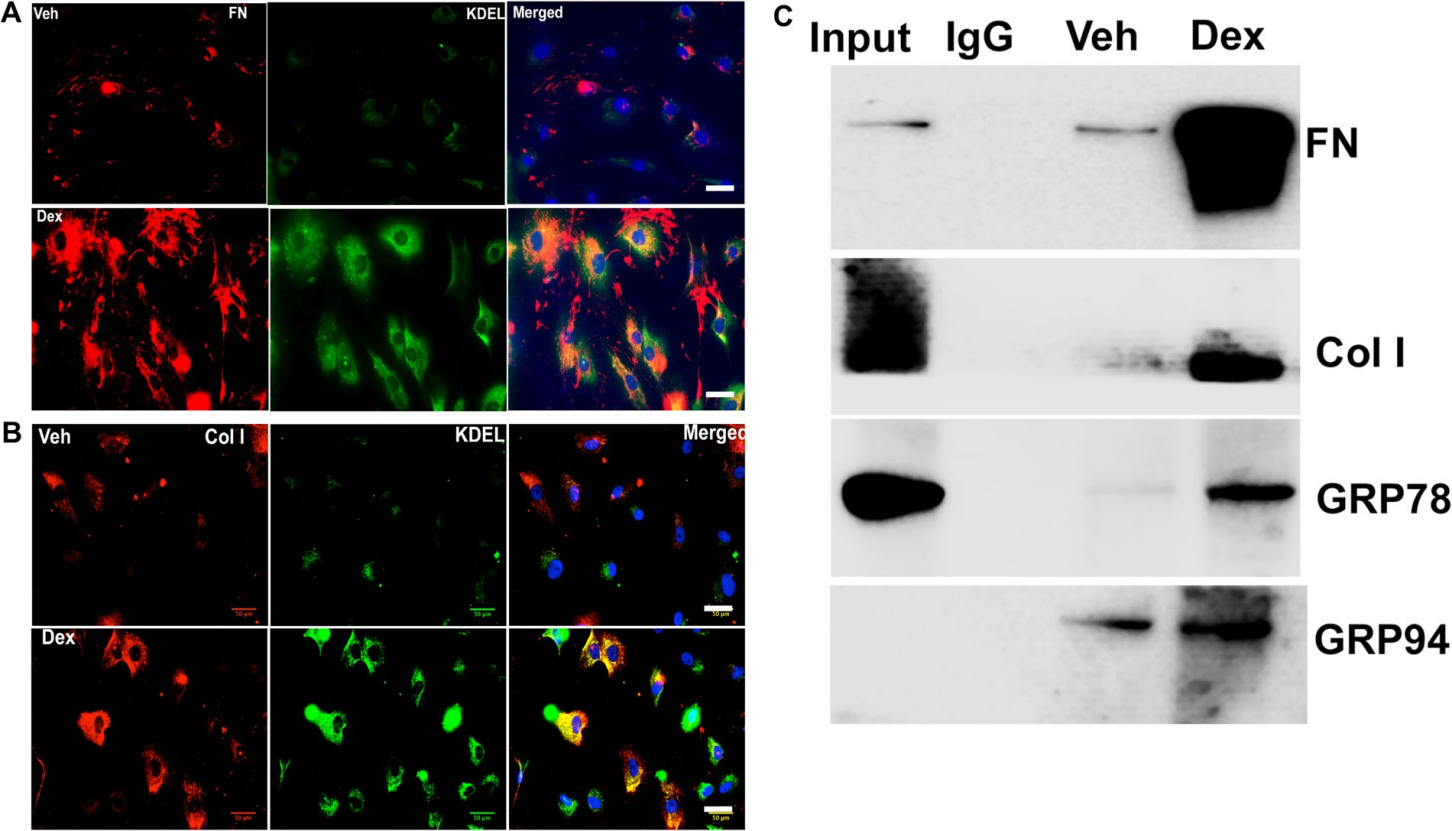
Dex induced ECM synthesis is associated with ER stress in TM cells. Primary human TM cells (n = 3 strains) were treated with Veh or Dex (100 nM) for 3 days and stained for KDEL and fibronectin (**A**) or Col I (**B**). A prominent co-localization of fibronectin or Col I with KDEL was observed in Dex-treated TM cells indicating induction of ER stress and excessive intracellular ECM overload in primary TM cells. (**C**) GTM3 cells were treated with Veh or Dex for 3 days and total lysates were subjected to immunoprecipitation using KDEL antibody and immunoblotted for ECM proteins, GRP78 and GRP94. IgG antibody was used as a negative control. Total cellular lysates were used as input.

To determine whether the Dex-induced excessive ECM protein load also induces ER stress in TM cells, we examined co-localization of ECM proteins with the ER stress marker KDEL. Both fibronectin and collagens constitute major ECM components of TM; therefore, we examined levels of fibronectin and collagen as general representatives of the trabecular meshwork ECM as described previously^53^. Increased co-localization of fibronectin (Fig. 4A) or Col I (Fig. 4B) with KDEL (ER stress marker) in Dex-treated cells demonstrated increased ECM synthesis and induction of ER stress in TM cells. We further examined whether Dex-induced fibronectin and Col I interact with ER stress proteins by performing co-immunoprecipitation studies (Fig. 4C). Total lysates were immunoprecipitated using KDEL antibody and blotted for ECM proteins. Lysates incubated with IgG antibody was used as a negative control and demonstrated no bands. A weak interaction of fibronectin and Col I with ER stress proteins (GRP78 and GRP94) was observed in Veh-treated cells, but this interaction increased prominently after Dex treatment. Note that although no band is observed for GRP94 in input samples at the low exposure shown in Fig. 3C, GRP94 band was observed in input samples at higher exposure. These data suggest that Dex-induced ECM synthesis is associated with ER stress in TM cells.

### Reduction of fibronectin prevents Dex-induced ER stress in TM cells

**Figure 5:**
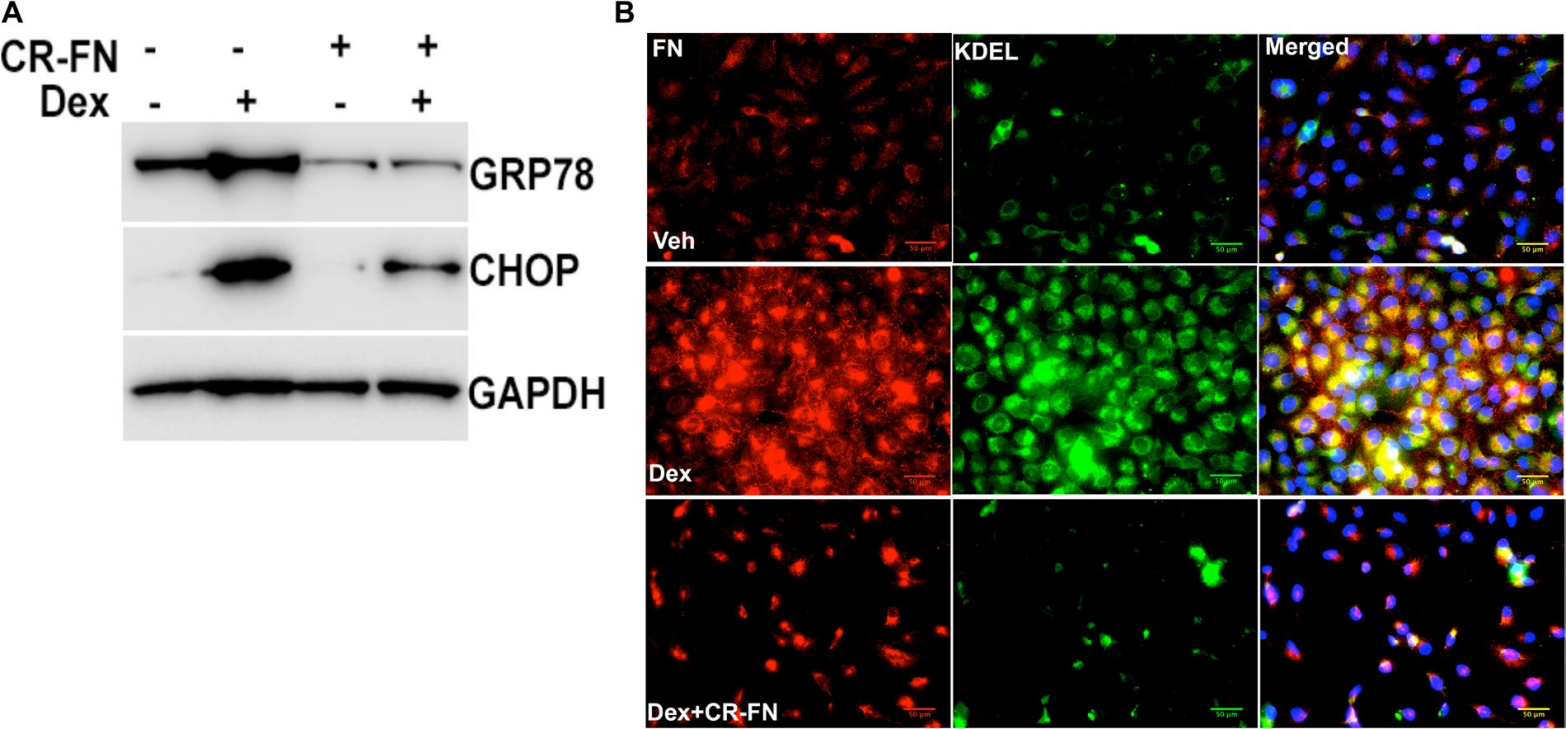
Reduction of fibronectin prevents Dex-induced ER stress in human GTM-3 cells. (**A**) GTM3 cells were transfected with a plasmid expressing CRISPR-Cas9 targeting fibronectin (CR-FN) and then treated with Veh or Dex for 48 hours. Fibronectin knockdown partially reduced ER stress as evident from reduced GRP78 and CHOP levels after Dex treatment (n = 2). (**B)** Human GTM3 cells were treated with Dex (100 nM) with or without CR-FN for 48 hours. Fixed cells were stained for fibronectin and KDEL to examine Dex-induced fibronectin and ER stress. Fibronectin knockdown reduced fibronectin and ER stress in Dex-treated TM cells (n = 2).

Previous studies have shown that fibronectin acts as a major regulator of ECM synthesis and deposition^54–56^. Therefore, we explored whether reduction of fibronectin prevents Dex-induced ER stress in GTM3 cells. GTM3 cells were transfected with or without CRISPR-Cas9 targeting fibronectin for 24 hours and then treated with Veh or Dex for 2 days. Knockdown of fibronectin in Dex-treated cells further reduced ER stress as evident from reduced levels of ER stress markers, GRP78 and CHOP (Fig. 5A). Immunostaining for fibronectin and KDEL revealed that fibronectin knockdown reduced fibronectin protein levels and also decreased ER stress in GTM3 cells treated with Dex (Fig. 5B).

### Cellular fibronectin induces ER stress in human TM cells

**Figure 6:**
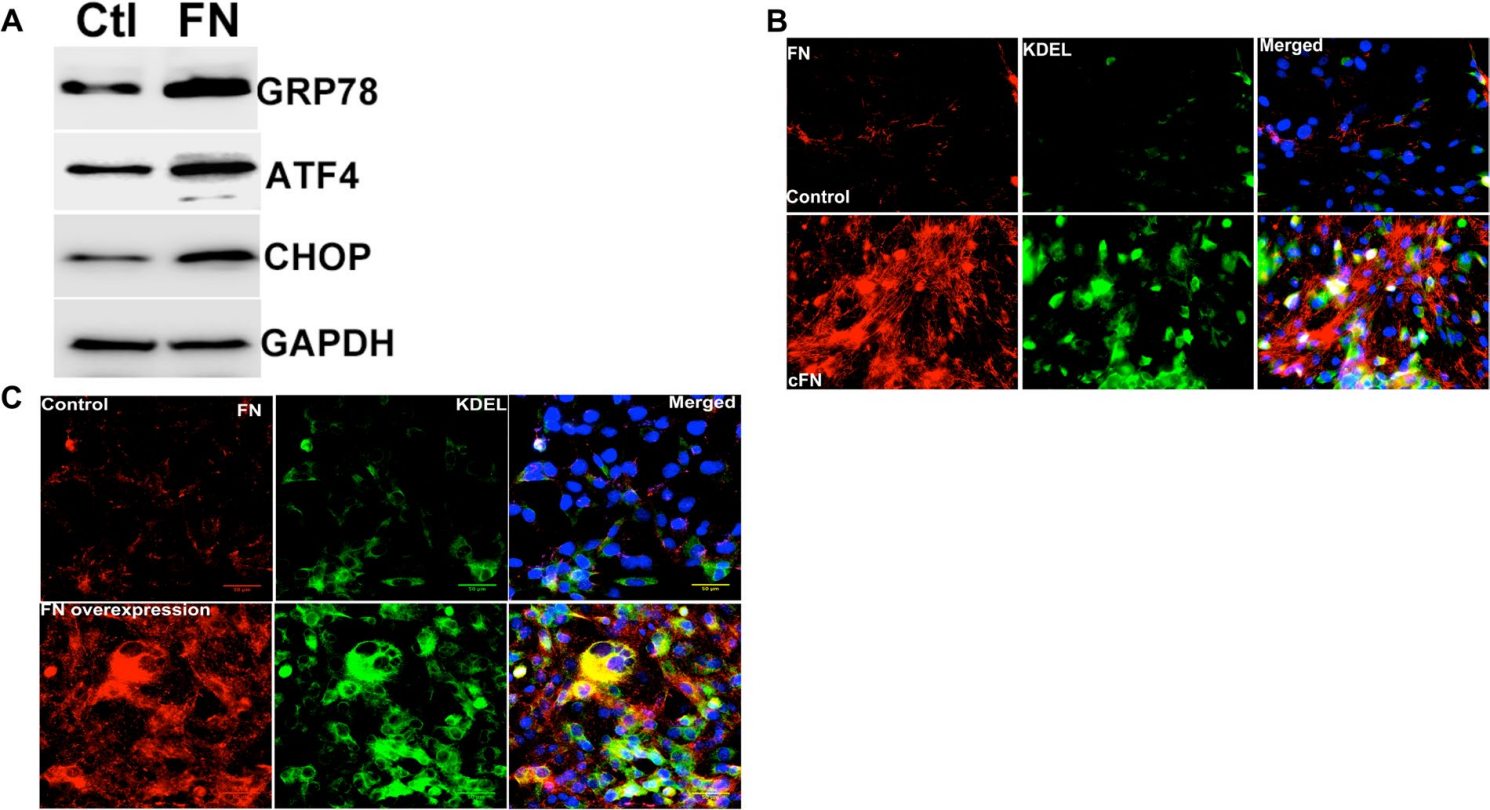
Overexpression of extracellular fibronectin induces ER stress in primary human TM cells. (**A**) Primary human TM cells (n = 2 strains) were treated with cellular fibronectin (10 ug/ml) for 48 hours and cellular lysates were analyzed for ER stress markers. (**B**) Primary human TM cells (n = 2 strains) were treated with cellular fibronectin (10 ug/ml) for 48 hours and fixed cells were stained with fibronectin and KDEL antibodies. Cellular fibronectin increased extracellular fibronectin and ER stress in GTM3 cells (n = 2). Scale bar = 50 μm. (**C**) GTM3 cells were transduced with fibronectin activation lentiviral particles for 48 hours and stained with fibronectin and KDEL antibodies. Increased fibronectin expression alone is sufficient to induce ER stress in TM cells (n = 2). Scale bar  =  50 μm.

Since increased extracellular ECM is known to be associated with human glaucomatous TM tissues, we next examined whether the extracellular deposition of ECM leads to ER stress in primary human TM cells. Cellular fibronectin is an insoluble fibronectin isoform that forms fibril networks and regulates ECM-cell interactions^57,58^. A recent study demonstrated that the cellular fibronectin isoform EDA was increased in post-mortem human glaucomatous TM tissues^7^. As shown in Fig. 6A, treatment of primary human TM cells with cellular fibronectin induced ER stress as evident from increased GRP78, ATF4, and CHOP further suggesting that extracellular deposition of ECM can induce ER stress in TM cells. Immunostaining showed increased extracellular fibronectin and ER stress induction in primary human TM cells treated with cellular fibronectin (Fig. 6B). Transduction of GTM3 cells with a lentiviral fibronectin expression vector also increased fibronectin and KDEL, indicating overexpression of fibronectin alone can induce ER stress in human TM cells (Fig. 6C).

### Decellularized Dex-induced extracellular ECM deposition is sufficient to induce ER stress in primary human TM cells

**Figure 7:**
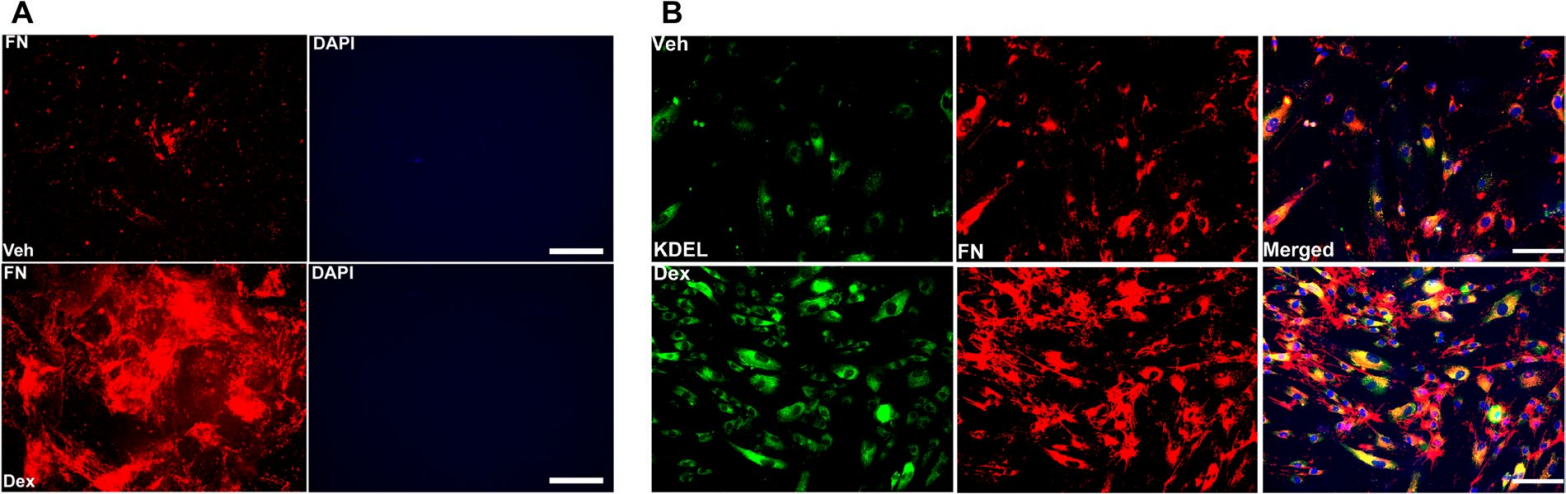
Dex-induced extracellular ECM leads to ER stress in primary human TM cells. (**A**) Primary human TM cells (n = 2 strains) were grown on chamber slides and treated with either Veh or Dex for 5 days. Upon decellularization, wells were stained with fibronectin and DAPI, to make ensure successful decellularization. An increased deposition of fibronectin is predominant in Dex treated cells compared to Veh treated cells. Absence of DAPI further ensures lack of cells after decellularization process. (**B)** Primary human TM cells (n = 2 strains) were grown on decellularized ECM for 5 days and stained with KDEL to detect ER stress. Induction of KDEL and fibronectin was observed in TM cells cultured on ECM derived from Dex-treated TM cells. Scale bar = 50 μm.

We next examined whether Dex-induced extracellular ECM leads to ER stress induction in human primary TM cells by culturing TM cells on ECM derived from Dex-treated TM cells. Human primary TM cells (n = 2 cell strains) grown in chamber slides were treated with Veh or Dex for 5 days. The ECM was decellularized from slides to remove TM cells. Immunostaining for fibronectin along with DAPI in decellularized slides demonstrated that Dex increased extracellular fibronectin staining while the absence of DAPI nuclear staining further supported successful decellularization (Fig. 7A). We also observed increased staining for Col I in Dex-treated ECM compared to Veh-treated ECM (data not shown). Fresh human primary TM cells were added to Veh or Dex-treated decellularized ECM and cells were cultured for an additional 5 days without any treatment (Fig. 7B). Interestingly, immunostaining revealed increased fibronectin and KDEL staining in TM cells cultured on the decellularized ECM from Dex treated cells, indicating that the Dex-induced extracellular ECM alone leads to ER stress in TM cells.

### TM cells are vulnerable to Dex-induced ER stress compared to other ocular cells

**Figure 8:**
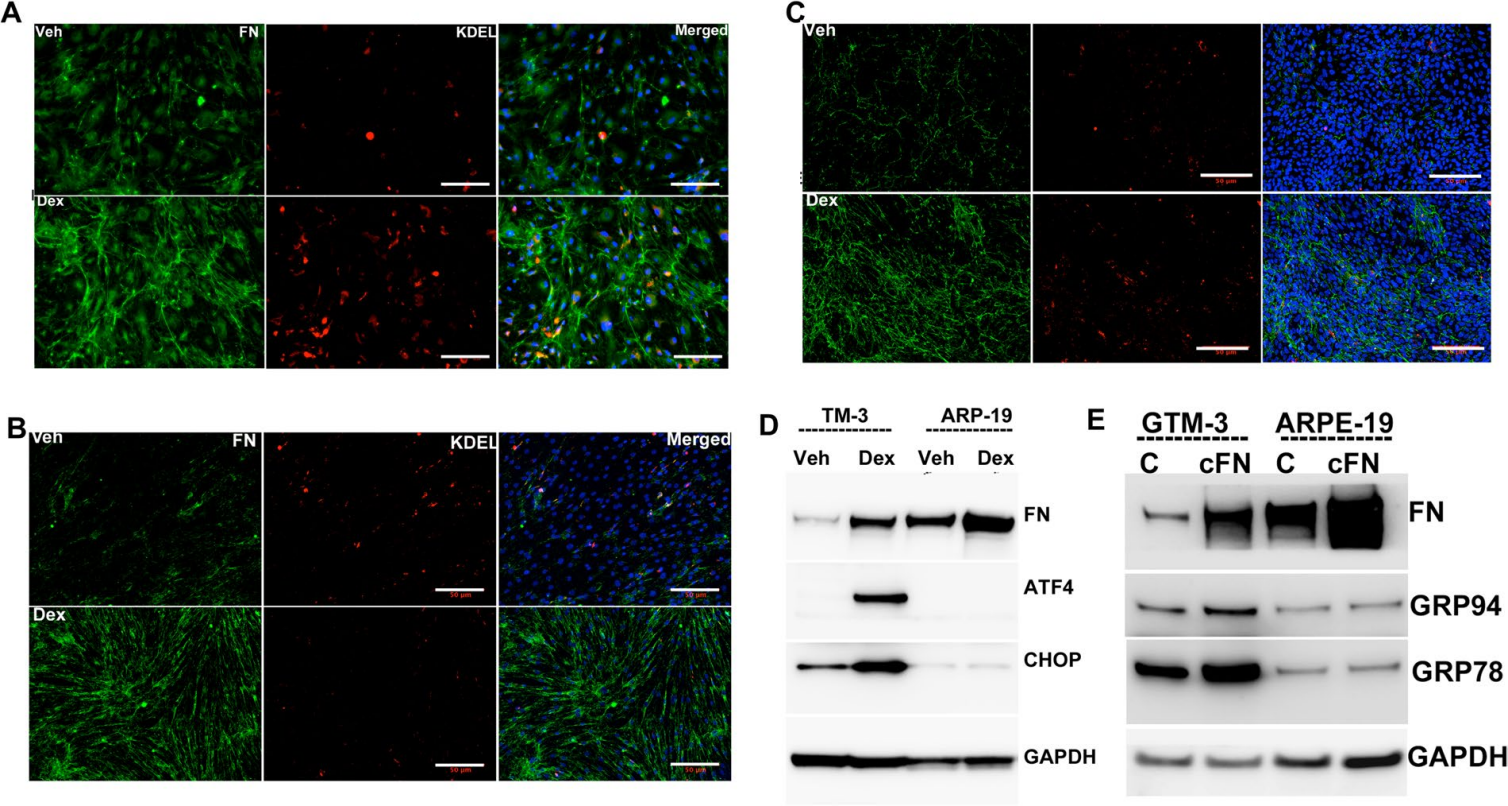
TM cells are sensitive to ER stress from Dex-induced abnormal ECM compared to other ocular cells: Primary human TM cells (**A**), RPE (**B**) and corneal fibroblasts (**C**) were treated with Veh or Dex for 3 days and immunostained for fibronectin and KDEL. Although fibronectin was increased in all of these cell types, ER stress was only observed in TM cells. (**D**) Human GTM3 or ARPE-19 cells were treated with Veh or Dex for 3 days. Fibronectin and chronic ER stress markers ATF4 and CHOP were examined in cellular lysates (n = 2). Although Dex increased fibronectin in both GTM3 and ARPE-19 cells, chronic ER stress was only observed in GTM3 cells indicating that TM cells are more prone to ER stress from Dex-induced ECM accumulation. (**E**) GTM-3 or ARPE-19 cells were treated with cellular fibronectin (cFn) for 2 days and ER stress was examined by Western blot analysis.

We further tested our hypothesis that TM cells are not well equipped to handle excessive ECM protein load. We explored whether TM cells are more susceptible to ER stress induction from Dex-induced synthesis and deposition of ECM proteins by comparing the ER stress response of different ocular cells to Dex treatment. Primary human TM, retinal pigment epithelial (RPE) cells and corneal fibroblasts (CF) were treated with Veh or Dex (100 nM) for 3 days and examined for Dex-induced ECM and ER stress (Fig. 8A–C). Immunostaining for fibronectin and KDEL demonstrated that Dex treatment increased fibronectin levels in all three-cell types at similar levels. However, ER stress induction was only evident in TM cells (Fig. 8A–C). We further examined chronic ER stress markers using Western blot analysis (Fig. 8D). Dex treatment increased fibronectin in both GTM3 and ARPE-19 cells. Interestingly, Dex treated GTM3 cells demonstrated induction of chronic ER stress markers including ATF4 and CHOP compared to Veh-treated cells. However, these markers were unaltered in ARPE-19 cells. Similarly, treatment of GTM3 or ARPE-19 cells with cellular fibronectin increased intracellular fibronectin but ER stress was only evident in GTM3 cells (Fig. 8E). These findings demonstrate that TM cells are more prone to ER stress induction from Dex-induced ECM synthesis and deposition.

### Increased levels and co-localization of fibronectin and Col I with ER stress marker in human glaucomatous TM tissues

**Figure 9:**
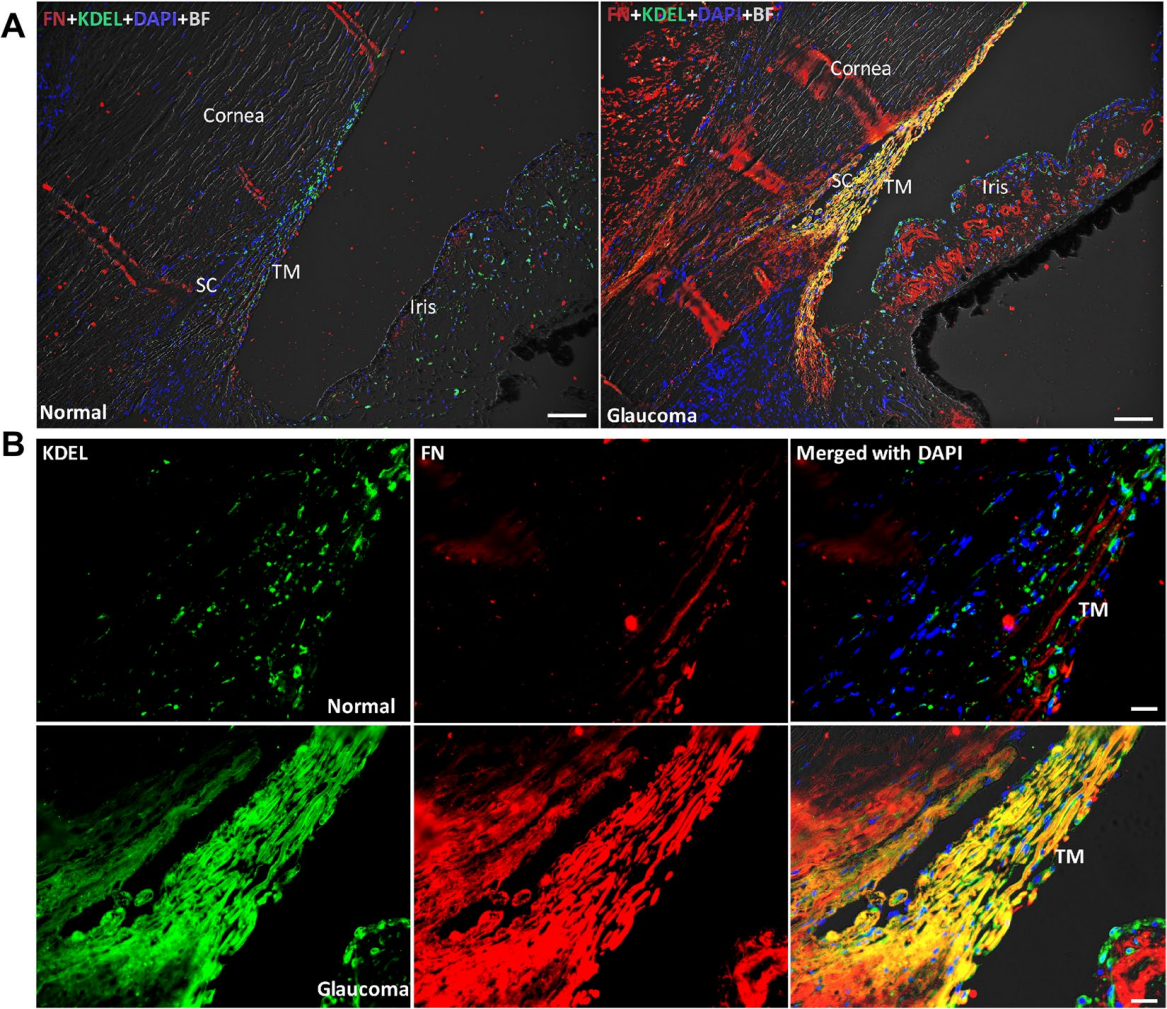
Increased co-localization of fibronectin with ER stress marker, KDEL in post-mortem human glaucomatous TM tissues. Post-mortem human TM tissues from age-matched normal (n = 6) and glaucoma (n = 6) were stained with fibronectin (green) and KDEL (red) and co-localization was examined in anterior segment tissues. Representative images are shown at lower (**A**) scale bar is 100 microns) and higher (**B**) scale bar is 25 microns) magnification. Both fibronectin and KDEL was increased in human glaucomatous TM tissues. In addition, increased co-localization of fibronectin with KDEL was observed in 5 out of 6 human glaucomatous TM tissues examined.

**Figure 10:**
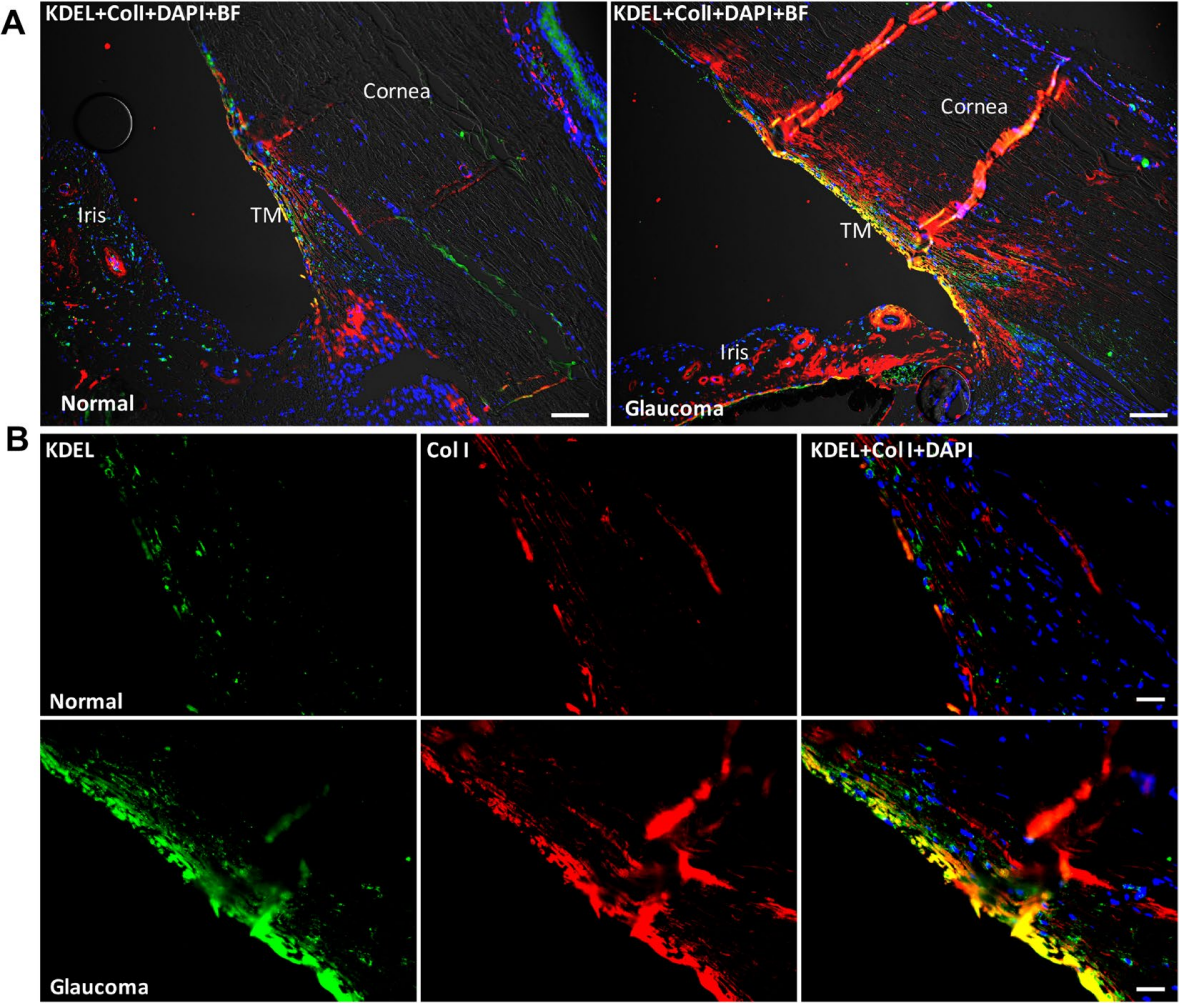
Increased co-localization of collagen type I with ER stress marker, KDEL in post-mortem human glaucomatous TM tissues. Post-mortem human TM tissues from age-matched normal (n = 3) and glaucoma (n = 3) were stained with Col I (green) and KDEL (red) and co-localization was examined in anterior segment tissues. Representative images are shown at lower (**A**) scale bar is 100 microns) and higher (**B**) scale bar is 25 microns) magnification. Both Col I and KDEL was increased in human glaucomatous TM tissues. In addition, increased co-localization of Col I with KDEL was observed in human glaucomatous TM tissues.

Since we observed increased co-localization of fibronectin with ER stress marker in primary human TM cells treated with Dex compared to Veh-treated TM cells, we next examined whether similar co-localization of fibronectin and Col I with ER stress is present in human glaucomatous TM tissues. Immunostaining for fibronectin and KDEL revealed that fibronectin and KDEL are noticeably increased in human post-mortem TM tissues from POAG donors compared to normal donor eyes (Fig. 9A,B and SI1). In addition, increased co-localization of fibronectin with KDEL was observed in these glaucomatous human TM tissues (Fig. 9A,B). Out of 6 pairs of human eyes immunostained, we observed a strong co-localization of fibronectin with KDEL in 5 glaucomatous eyes. Similarly, we observed increased Col I and its co-localization with ER stress markers in all glaucomatous TM tissues compared to age-matched normal donor tissues (Fig. 10A,B and SI2). Importantly, we observed that both ECM and ER stress markers were consistently increased in all glaucomatous TM tissues (SI1 and SIII). These studies suggest that increased ECM accumulation is associated with ER stress in the glaucomatous TM tissues.

## Discussion

Excessive ECM accumulation is considered a major feature of the glaucomatous TM tissues. However, it is not yet understood how this ECM accumulation leads to TM dysfunction and IOP elevation. Here, we demonstrate that GC-induced ocular hypertension and reduced outflow facility is associated with increased ECM accumulation in the TM. We further demonstrate that Dex increases secretory protein load of ECM proteins, inducing ER stress in primary human TM cells. Dex treatment increases interactions between the ER stress marker GRP78 with fibronectin and Col I in TM cells. Reduction of fibronectin prevents ER stress in Dex-treated TM cells. The expression of cellular fibronectin and ER stress markers is higher in the TM of open angle glaucoma eyes^7,49^; treatment of cultured primary TM cells with exogenous cellular fibronectin also induced ER stress in cultured primary TM cells. Interestingly, ECM derived from Dex-treated cells was sufficient to induce ER stress in normal TM cells. We further demonstrate that TM cells are sensitive to ER stress from increased ECM protein accumulation compared to other ocular cell types. Importantly, increased levels of fibronectin and Col I and their co-localization with an ER stress protein was observed in human glaucomatous TM tissues further suggesting that increased synthesis and deposition of ECM is associated with ER stress in the TM.

A recent study demonstrated that systemic Dex treatment in mice leads to reduced outflow facility and increased fingerprint-like materials in the TM^42^. Similar ECM changes were also observed in TM tissues of humans treated with steroids^20,22,23,59^. Consistent with these findings, we show that GC-induced ocular hypertension is associated with increased aqueous humor outflow resistance and increased ECM accumulation in the mouse TM. Therefore, it is likely that Dex-induced abnormal ECM deposition in the TM is responsible for this increased outflow resistance.

Treatment of human TM cells with cellular fibronectin induced chronic ER stress proteins including ATF4 and CHOP, which are known to be associated with cell dysfunction/death. We have previously demonstrated increased ATF4 and CHOP levels in the glaucomatous TM tissues from POAG patients^49^ and in anterior segment tissues of mouse models of myocilin or GC-induced glaucoma^41,60^. Chronic ER stress can cause detrimental effects on TM function. In addition, the overwhelmed ER may compromise the quality of secreted ECM proteins, which are more likely to form abnormal depositions in the ECM as observed in human TM tissues from GC-induced glaucoma and POAG donors. Consistent with this, we have recently demonstrated that ER stress induced by mutant myocilin causes intracellular retention of selected ECM proteins^53^.

Although we focused our studies to selected ECM proteins, it is likely that accumulation of other ECM proteins is also associated with ER stress in the TM. Dex has been shown to induce expression of several ECM proteins^14,16,23^, which are normally processed in the ER. Consistent with this, we observed that Dex increases de novo protein synthesis of secretory proteins, including selected ECM proteins. We observed increased load of fibronectin and Col I in the ER of TM cells, which may further compromise ER homeostasis, inducing ER stress in TM cells. Genetic knock down of fibronectin alone was able to partially prevent Dex-induced ER stress. Since we hypothesize that Dex-induced ER stress is result of a cumulative load of several ECM proteins that are increased by Dex, a single knock down of ECM proteins other than fibronectin may not abrogate Dex-induced ER stress. We chose fibronectin knock down for multiple reasons. First, fibronectin is one of the predominant ECM protein in the TM and it is prominently induced by Dex. Second, fibronectin is known to regulate the assembly of other ECM proteins in TM cells. A recent study has shown that disruption of fibronectin matrix prevents Dex-induced deposition of collagen type IV, fibrillin and laminin into ECM of TM cells^54^. This would result in overall decrease in Dex-induced overload of other ECM proteins, thereby preventing ER stress in TM cells. Third, fibronectin was increased and co-localized to ER stress proteins in the human glaucomatous TM tissues. To evaluate the effects of other ECM on ER stress, we have utilized the ECM derived from Dex-treated cells. Primary TM cells grown on ECM derived from Dex-treated cells exhibited ER stress indicating that Dex-induced extracellular ECM is sufficient to induce ER stress. Nonetheless, it should be noted that our study is limited to examining the effects of fibronectin alone in Dex-induced ER stress.

It is likely that Dex-induced excessive intracellular ECM load is a result of increased synthesis load, decreased proteosomal degradation, and inefficient secretion. In addition, our studies demonstrate that increased extracellular fibronectin deposition alone is sufficient to induce ER stress in TM cells as evident from the following findings. First, treatment of human primary TM cells with cellular fibronectin, which forms extracellular fibronectin deposits induces ER stress. Second, induction of ER stress was evident when normal TM cells were grown on decellularized ECM from Dex-treated cells. These findings are important for POAG pathology since several studies have shown extracellular deposition of ECM material^14,23^ as well as cellular fibronectin^7^ in glaucomatous TM tissues. Increased co-localization of fibronectin with ER stress in human TM tissues from POAG donor eyes further supports that ER stress is associated with increased ECM in the TM. It is interesting to note that ER stress induction was only observed in the TM region of POAG donor eyes despite the increase in both fibronectin and Col I in other regions of anterior segment tissues. This further supports our hypothesis that TM is more sensitive to ER stress from ECM accumulation.

To our knowledge, this is first study that demonstrates induction of ER stress from increased ECM accumulation. It is not clear how TM cells communicate with the ECM to induce ER stress. Cells can communicate with ECM via fibronectin-integrin interactions to regulate cellular responses^61–63^. Fibronectin is known to act as major regulator of ECM assembly^56,64^. Therefore, fibronectin may act as a major regulator of Dex-induced ECM accumulation and ER stress induction in TM cells. Consistent with this, a recent study has demonstrated that treatment of primary human TM cells with cellular fibronectin increased expression of other ECM proteins including collagen I and laminin^65^. In the present study, we observed that knockdown of fibronectin reduced Dex-induced ER stress in TM cells. Moreover, treatment of TM cells with cellular fibronectin was sufficient to induce ER stress. It is therefore likely that fibronectin regulates several other ECM proteins in the TM. It is interesting to note that TM cells are more sensitive to ER stress from Dex-induced ECM accumulation compared to other ocular cells. Considering the unique function of TM cells, it is possible that TM cells are incapable of handling increased protein accumulation, inducing chronic ER stress proteins such as CHOP, which may further exacerbate chronic ER stress.

In conclusion, we demonstrate that increased ECM synthesis and deposition leads to ER stress in the TM, which may be associated with TM dysfunction and IOP elevation. We further present evidence that TM cells are more sensitive to ER stress from Dex-induced ECM accumulation. Importantly, ER stress is associated with increased ECM accumulation in human TM tissues from POAG donors. It is possible that targeting ER stress pathway may provide strategies to reduce ECM accumulation in the glaucomatous TM.

## Materials and Methods

### Antibodies

Antibodies were purchased from the following sources: fibronectin (catalog # Ab2413, Abcam, Cambridge, MA, USA), KDEL (catalog # Ab12223, Abcam, Cambridge, MA, USA), collagen I (catalog # NB600-408, Novus Biologicals, Littleton, CO, USA), ATF-4 (catalog # SC-200, Santa Cruz Biotechnology, Dallas, TX, USA)), CHOP (catalog # 13172, Novus Biologicals, Littleton, CO, USA), Alexa Fluor® phalloiden stain (catalog # A12379, Life technologies, Grand Island, NY, USA), GRP78 (catalog # ab21685, Abcam, Cambridge, MA, USA), α-smooth muscle actin (catalog #ab7817, Abcam, Cambridge, MA, USA) and GAPDH (catalog # 3683, Cell signaling technology, Danvers, MA, USA). Both KDEL and GRP78 were used as ER stress markers. Mouse KDEL antibody was primarily used for immunostaining since it works well on fixed cells and formalin fixed human tissues and can be combined with rabbit fibronectin or collagen I antibody. We observed that KDEL primarily recognizes GRP78 and GRP94 in Western blot analysis of human TM cells. Therefore, we used KDEL as an ER stress marker. Rabbit GRP78 antibody was used along with mouse puromycin antibody for immunostaining. For Western blot analysis of ER stress in cells, we primarily utilized the GRP78 antibody.

### Experimental Animals

A/J mice were obtained from the Jackson Laboratory (Bar Harbor, ME). Animals were fed standard chow *ad libitum* and housed in 12h light/12h dark conditions. All experimental procedures were conducted in accordance with and adherence to the ARVO Statement for the Use of Animals in Ophthalmic and Vision Research, and experimental protocols were approved the University of North Texas Health Science Center (UNTHSC) Institutional Animal Care and Use Committee (IACUC) Regulations and Guidelines (protocol approval # IACUC-2015-0002).

### Dex treatment of mice

Topical 0.1% Dex phosphate (Bausch & Lomb Inc.) or Veh eye drops containing sterile phosphate-buffered saline (PBS) were applied to 3-month-old A/J mice thrice daily for 7 weeks (Fig. 1) as described previously^41^. At the end of the treatment, whole eyes were fixed & sectioned for immunostaining.

### Intraocular Pressure (IOP) Measurement

Intraocular pressure (IOP) was determined in behaviorally trained conscious mice using a TonoLab rebound tonometer (Colonial Medical Supply, Franconia, NH, USA) as previously described^66^. Day-time IOPs were measured between 10 AM to 2 PM. Approximately an average of 6 individual IOP measurements were taken to calculate the final IOP value for each eye. All IOP measurements were recorded in a masked manner.

### Aqueous Outflow Facility (C)

Aqueous outflow facility (C) was measured in 7-week Dex-treated mice by constant flow infusion as previously described^67,68^. Briefly, animals were anesthetized (ketamine 100 mg/kg; xylazine 10 mg/kg, I.P.), and the tip of a 30G steel needle was carefully placed into the anterior chamber. The needle was connected by tubing to a flow-through pressure transducer (BLPR2, World Precision Instruments (WPI), Sarasota, FL USA). The opposing end of the transducer was connected to a 1mL syringe filled with sterile filtered PBS loaded into an infusion pump (Microdialysis SP101i, WPI). A virtual chart recorder (LabScribe2, WPI) continuously displayed pressure signals. Following equilibration (20–30 min), eyes were infused at a flow rate of 100 nL/min. When pressure had stabilized (10–15 min), flow rates were increased sequentially (200 to 500 nL/min, in 100 nL/min increments). Three stabilized pressures at each flow rate were recorded, and the average value calculated as mean stabilized pressure. C was calculated as the reciprocal of the slope of a plot of Mean Stabilized Pressure as the ordinate and Flow Rate the as abscissa.

### Dex treatment of human TM cells

Four different primary human TM cell strains and a transformed GTM3 cell line were cultured in DMEM medium (Sigma, St. Louis, MO, USA) supplemented with 10% fetal bovine serum (Atlas Biologicals, Fort Collins, CO, USA), L-glutamine (Gibco, Life technologies, Grand Island, NY, USA), and Pen-strep (Gibco, Life technologies, Grand Island, NY, USA). For characterization of primary human TM cells, cells were examined for the expression of fibronectin, collagen and laminin as well as Dex-induction of crosslinked actin networks and myocilin as described previously^69–71^. TM cells were treated with either Veh (0.1% ethanol) or 100 nM Dex (Sigma-Aldrich Corp., St. Louis, MO, USA) for various time periods in serum-free conditions. For Western blot analysis, both conditioned medium and cellular lysates were collected. For immunostaining, cells were fixed and stained with appropriate antibodies. To examine the effect of fibronectin knockdown on Dex-induced ER stress, GTM3 cells were transfected with a fibronectin CRISPR/Cas9 KO plasmid (SC-40082, Santa Cruz Biotechnology, Dallas, TX) using Lipofectamine 3000 (Life Technologies, Grand Island, NY, USA). 24 hours after transfection, cells were treated with Veh or Dex for another 2 days. Cell lysates were utilized for Western blot analysis while fixed cells were immunostained to detect fibronectin and KDEL. The effect of fibronectin overexpression on ER stress was examined by transducing GTM3 cells with lentiviral fibronectin expression particles (SC-400082-LAC, Santa Cruz Biotechnology, Dallas, TX) for 2 days according the manufacturer instructions. Cells were fixed and immunostained with fibronectin and KDEL antibodies. For treatment with cellular fibronectin, human primary TM cells (n = 2 cell strains) were treated with 10 ug/ml cellular fibronectin (F2518; Sigma-Aldrich Corp., St. Louis, MO, USA) for 48 hours; cell lysates or fixed cells were analyzed for ER stress.

### Decellularization

Decellularization was performed as described previously^72,73^. Briefly, primary human TM cells (n = 2 strains) were grown on 4-well chamber slides. After reaching approximately 70% confluency, cells were treated with either Veh or Dex (100 nM) for 5 days. Following the treatment, cells were detached by treating with 2 mL of 0.2% Triton X-100 in water at 37 °C for 10 min and detached cells were washed off with 1X PBS, (3 times). 2 mL of 0.3% ammonium hydroxide solution was added slowly to the wells and incubated for 5 min at 3 °C. Nuclear staining with DAPI was done to ensure a complete decellularization, and increased deposition of ECM was confirmed by immunostaining for fibronectin. Following the complete decellularization process, untreated primary human TM cells were re-plated on the same 4-well chamber slides and cultured to confluence. Immunostaining was used to analyze the ER stress (KDEL) in the TM cells induced by the decellularized ECM.

### Effect of Dexamethasone on different ocular cells

Primary human TM cells as well as primary human corneal fibroblasts and primary human retinal pigment epithelium cells (obtained from ScienCell Research laboratories, Carlsbad, USA) were grown on 4-well chamber slides. After reaching an approximately 70% confluence, cells were treated with either Veh or Dex (100 nM) for 3 days. Dex- induced ECM (FN) and ER stress (KDEL) were analyzed by immunostaining. Transformed GTM3 and ARPE-19 cells were grown to approximately 70% confluence in 6-well plates and treated for 3 days either with Veh or Dex or cellular fibronectin (10 ug/ml). Following the treatment, medium was removed and cells were washed with 1X PBS and lysed using 1X lysis buffer, supplemented with 1X protease inhibitor cocktail. Cell lysates were subjected to Western blotting to analyze ECM (FN) and ER stress (KDEL and CHOP) markers. GAPDH was used as the loading control.

### Immunostaining

Mouse eyes from Veh or Dex-treated groups were enucleated and fixed in freshly prepared 4% PFA for 3 hours, cryo-protected by keeping fixed eyes in 30% sucrose overnight before OCT embedding. 10 µ thin sections were made using a cryostat (Leica Inc, Buffalo Grove, IL, USA). The sections were allowed to dry at room temperature prior to use. The sections were then permeabilized using 0.1% Triton-X-100 in PBS for 15 min and blocked with 10% normal goat serum for an hour. Slides were incubated overnight with primary antibody (1:100 dilution) in 10% (v/v) normal goat serum, and then washed 3 times with PBS followed by 2-hour incubation with appropriate Alexa Fluor secondary antibodies (1:200; Life technologies, Grand Island, NY, USA). Sections were mounted with DAPI-mounting solution. Images were captured using Keyence microscope (Itasca, IL, USA). Cultured TM cells were fixed in 4% PFA for 15 minutes and permeabilized with 0.1% Triton-X-100 in 1xPBS for 10 minutes followed by 30 minutes blocking with 10% goat serum. The cells were then incubated with appropriate primary & secondary antibodies similarly as described above.

### Western blot analysis

The protein samples (30 ug total protein) were run on denaturing 4–12% gradient polyacrylamide ready-made gels (NuPAGE Bis-Tris gels, Life technologies, Grand Island, NY, USA) and transferred onto PVDF membranes. Blots were blocked with 10% non-fat dried milk for 1 hour then incubated overnight with specific primary antibodies at 4 °C on a rotating shaker. The membranes were washed thrice with PBST and incubated with corresponding HRP-conjugated secondary antibody for 90 minutes. The proteins were then visualized using ECL detection reagents (SuperSignal West Femto Maximum Sensitivity Substrate; Life technologies, Grand Island, NY, USA).

### Surface sensing of translation (SUnSET) Assay

GTM3 cells were grown to approximately 70% confluence in 6-well tissue culture plates. Cells were treated with either vehicle control (ethanol, 0.1%) or Dex (100 nM) in serum free medium for 3 days. Cells without any treatment acted as a control. Following 3 days, one of the controls was treated with cycloheximide (10 ug/ml) for 16 hrs. The conditioned medium was removed from all the wells and incubated with serum free medium containing puromycin (10 ug/ml) for 30 min as described previously^52^. Following 30 min incubation, puromycin containing medium was removed and cells were washed twice with 1x PBS. Cells were lysed with ice cold 1x lysis buffer, containing protease and phosphatase inhibitors cocktail. The obtained cell lysates were subjected to Western blot analysis. Rabbit Anti puromycin antibody (1:10,000, Sigma) was used as the primary antibody and goat anti rabbit-HRP conjugated was used as the secondary antibody for analyzing puromycin incorporated into the newly synthesized proteins. Actin on the same blot was used as loading control and Coomassie brilliant blue staining was performed to examine total proteins.

### Co-immunoprecipitation

GTM3 cells were treated with Veh or Dex for 3 days and lysates were immunoprecipitated with anti-KDEL antibody using Dynabeads™ Co-Immunoprecipitation Kit (Life technologies, Grand Island, NY, USA). KDEL or IgG antibodies (2.5 ug) were diluted in 200 ul of lysis buffer and incubated with 50 ul of Dynabeads protein G for 10 minutes at room temperature. The beads-Ab complexes were washed with lysis buffer 3 times according to manufacturer protocol using a magnet separator. Total protein lysates (100 ug) were added to the above complexes for 10 minutes at room temperature. The beads-Ab-Ag complexes were then washed 3 times with lysis buffer containing 0.5% Triton-x-100 and precipitated proteins were analyzed by SDS/PAGE and Western blotting following our standard protocol. Note that cell lysates from Veh-treated cells (10 ug) was loaded in SDS/PAGE gel as an input.

### Immunohistochemistry of human TM tissues

Age-matched (between 70–80 yrs) normal (n = 6) and POAG (n = 6) donor eyes were stained for fibronectin, Col I and the ER stress maker KDEL as described previously^49^. The eyes were obtained and managed in compliance with the Declaration of Helsinki. Briefly, eyes were obtained from the Lions Eye Institute for Transplant and Research (Tampa, FL) within 6 hours of death and fixed in 10% formalin. This is an accredited eye bank where donor or family permission is received and the eye tissues can be used for transplant or research. The eye tissues were fixed at the Lions Eye Institute before sending to our lab. The slides containing the anterior segment sections were deparaffinized in xylene and dehydrated twice with 100%, 95%, 70%, 50% ethanol for 5 minutes each. It should be noted that these sections were not subjected to antigen retrieval because we observed strong fibronectin staining in the cornea that obscured staining in TM region when sections were processed in boiling citrate buffer. Sections were incubated with 1% Triton containing 10% goat serum for 2 hours. Sections were incubated overnight at 4 °C with mouse KDEL and rabbit fibronectin or collagen I antibodies (1:300 in blocking buffer). Slides were washed with PBS and incubated with Alexa donkey-anti-rabbit 568 and Alexa donkey anti-mouse 488 (1:500 in PBS, Thermo Fisher Scientific Inc., Pittsburgh PA) for 2 hrs. Sections were washed with PBS and mounted with mounting medium containing DAPI (Vector Labs, Inc. CA). Sections incubated with no primary antibody served as a negative control.

## Electronic supplementary material

SI
