# Supplementary material for: Increased synthesis and deposition of extracellular matrix proteins leads to endoplasmic reticulum stress in the trabecular meshwork
Source: Sci Rep. 2017 Nov 2;7:14951. doi: 10.1038/s41598-017-14938-0 (PMC5668243; doi:10.1038/s41598-017-14938-0)
Supplement: Supplementary file 1 — SI [file 41598_2017_14938_MOESM1_ESM.doc]

**Increased synthesis and deposition of extracellular matrix proteins leads to endoplasmic reticulum stress in the trabecular meshwork**

Ramesh B. Kasetti^1^, Prabhavathi Maddineni^1^, J. Cameron Millar^1^, Abbot F. Clark^1^, and Gulab S. Zode^1^

**Supplementary Information**

**
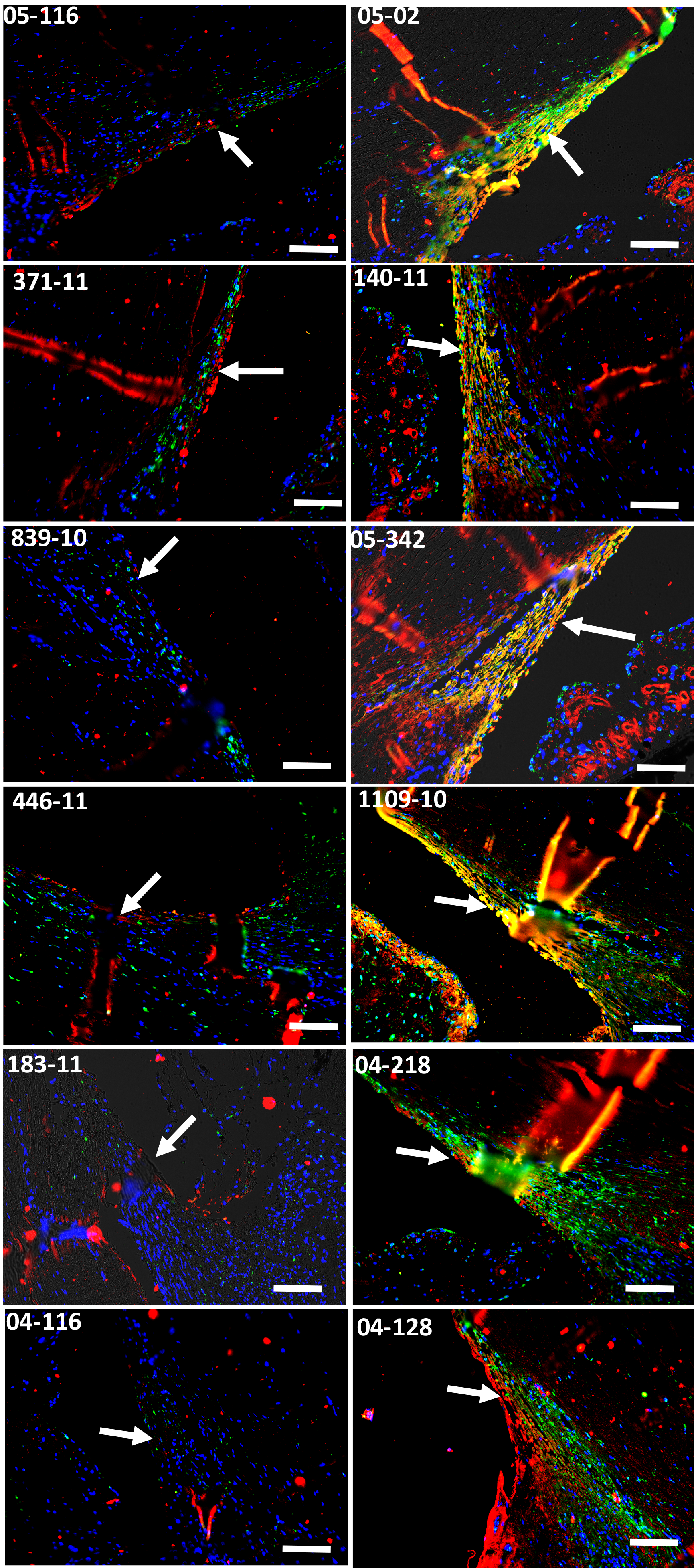
**

**SI:1: FN and KDEL staining in human post-mortem TM tissues:** Age-matched normal and glaucoma donor human anterior segment tissues were stained for fibronectin (red) and KDEL (green). Images were taken using 20x objective and merged (FN+KDEL+DAPI) images of all eyes are shown. Scale bar is 100 microns. Arrow shows TM region. TM tissues from all 6 normal donor eyes are shown on left panel and TM tissues from POAG donors are shown on right panel. Increased immunostaining for fibronectin and KDEL as well as increased co-localization was observed in TM tissues of all glaucoma donors.

**
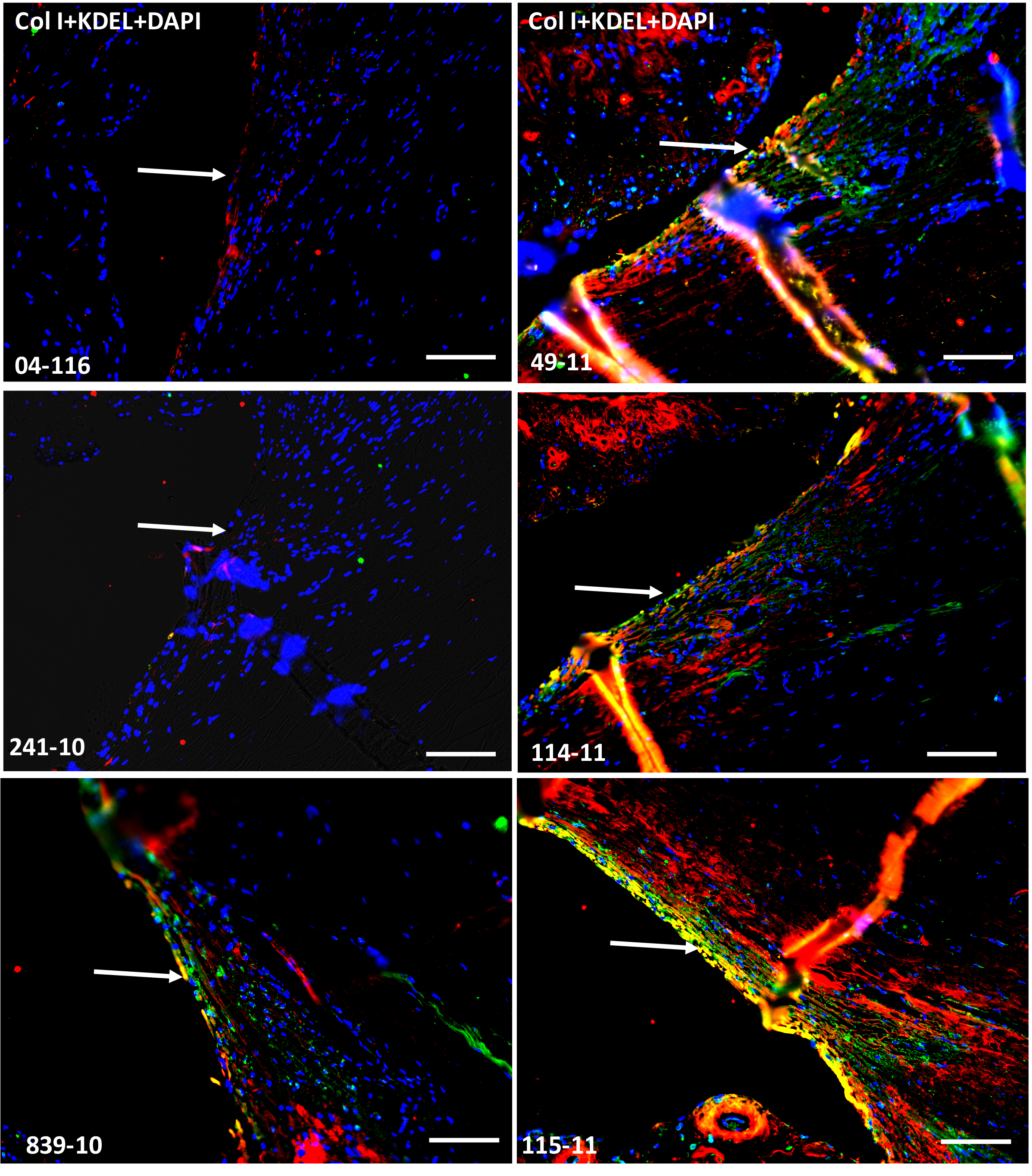
**

**SI:2: Collagen I and KDEL immunostaining in human post-mortem TM tissues:** Age-matched normal and glaucomatous anterior segment tissues were stained for fibronectin (red) and KDEL (green). Images were taken using 20x objective and merged (Col I+KDEL+DAPI) images of all eyes are shown. Scale bar is 100 microns. Arrow shows TM region. TM tissues from normal eyes are shown on left side and TM tissues from POAG donors are shown in right side. Increased immunostaining for collagen type I and KDEL as well as increased co-localization was observed in TM tissues of all glaucoma donors.
